# Editorial: Challenges in pediatric endocrinology regarding alterations in glucose metabolism, growth disorders, disorders of sex development, and puberty in adolescents

**DOI:** 10.3389/fped.2025.1561951

**Published:** 2025-02-03

**Authors:** Giulio Maltoni, Egidio Candela, Gianluca Tornese

**Affiliations:** ^1^ Pediatric Unit, IRCCS Azienda Ospedaliero-Universitaria di Bologna, Bologna, Italy; ^2^ Department of Medical and Surgical Sciences, Alma Mater Studiorum – University of Bologna, Bologna, Italy; ^3^ Institute for Maternal and Child Health IRCCS “Burlo Garofolo”, Trieste, Italy; ^4^ Department of Medicine, Surgery and Health Sciences, University of Trieste, Trieste, Italy

**Keywords:** central precocious puberty (CPP), type 1 diabetes mellitus, puberty timing, microbiome, early pubertal development, Sirtuin

**Editorial on the Research Topic**
Challenges in pediatric endocrinology regarding alterations in glucose metabolism, growth disorders, disorders of sex development, and puberty in adolescents

While a relatively established field of study, pediatric endocrinology has witnessed a significant acceleration in acquiring advanced and nuanced knowledge in recent years. This rapid progress stems from breakthroughs in genetics, integration of machine learning, enhanced understanding of the microbiome and human biology, advancements in laboratory methodologies, and novel therapeutic options (1, 2).

Building on this line of study, the present research topic has concentrated on applying this integrative approach to studying idiopathic central precocious puberty (CPP) and type 1 diabetes mellitus (T1DM), two well-recognized conditions that remain incompletely understood.

An excellent example of a new frontier is the issue addressed by the meta-analysis of Lan et al., whose results highlight the high diagnostic accuracy achieved by machine learning models utilizing clinical, hormonal (laboratory), and imaging data for diagnosing CPP, with impressive sensitivity, specificity, and area under the curve (AUC) values, showcasing their potential for improving diagnostic precision.

Genetic influences on puberty timing are also gaining prominence. For instance, an extensive multi-ancestry genetic analysis of ∼800,000 women identified 1,080 genetic signals associated with age at menarche, suggesting that while genetically driven CPP remains a minority, the identification of genetic role in timing is increasing (3, 4).

The study by Li et al. is particularly noteworthy. It developed an integrated predictive model for early pubertal development (EPD) in girls, incorporating genetic factors (28 single-nucleotide polymorphisms, SNPs) and non-genetic factors (e.g., overweight, and lifestyle habits). Interestingly, non-genetic factors proved to be more impactful in prediction, indicating the need for a holistic approach to assessing EPD risk.

Equally intriguing is the work conducted by Ying et al., which assessed the effects of mechanistic targets of rapamycin (mTOR)- dependent circulating protein levels on CPP. Mendelian randomization analyses identified a causal association between plasma levels of one of these proteins, eIF4G, and CPP onset, suggesting its potential as a biomarker for prevention or treatment.

The study by Jiang et al. explored clinical aspects of CPP less commonly studied, such as ophthalmic evaluations. Using the Objective Scatter Index (OSI), the identified significant alterations to estrogen levels, opening new avenues for research into the systemic effects of CPP.

The research on T1DM highlights another critical area of pediatric endocrinology. For example, a study by Wang et al. revealed distinct microbiological characteristics in oropharyngeal and gut microbiota in children with T1DM, including alterations in metabolic pathways related to amino acid and fatty acid metabolism. These findings are particularly relevant given a recent meta-analysis showing that children and adolescents with T1DM who received probiotics exhibited poorer glycemic control than controls. This is a counterintuitive result that warrants further investigation into the complex interplay between microbiome and glycemic outcomes (5).

The review by Lan et al. takes a broader perspective, focusing on the age-specific challenges of T1DM management, emphasizing the interaction between physical, psychological, and social factors. The review proposes practical strategies tailored to different developmental stages, offering actionable insights for clinicians.

Fedorczak et al. study explored the role of sirtuin (SIRT1) in growth and metabolism. Their findings indicate that dietary factors, such as fruit and vegetable intake, influence SIRT1 more significantly than biological variables, like age or pubertal stage. This highlights the interplay between nutrition, metabolic health, and growth, providing a foundation for future research.

The studies presented in this Research Topic illustrate how leveraging multidisciplinary approaches, including genetics, machine learning, and microbiome research, can drive significant advances in pediatric endocrinology. These findings emphasize the need for personalized strategies and multidisciplinary collaboration to address the complexities of conditions like CPP and T1DM. Further research is required to validate emerging biomarkers and integrate novel technologies into clinical practice, paving the way for improved diagnostic and therapeutic outcomes.
